# “You’ve involved us knowing that we don’t think about the law like you”: a qualitative study on Supersavers’ perspective of overdose engagement in Skåne county, South Sweden

**DOI:** 10.1186/s12954-026-01487-x

**Published:** 2026-06-23

**Authors:** Katja Troberg, Pernilla Isendahl

**Affiliations:** 1 Malmö Addiction Centre, Clinical Research Unit, Skåne University Hospital, Region Skåne, Södra Förstadsgatan 35, plan 4, Malmö, 205 02 Sweden; 2 Department of Clinical Sciences Lund, Psychiatry, Faculty of Medicine, Lund University, Sölvegatan 19, Lund, 221 84 Sweden; 3 Department of Infectious Disease, Skåne University Hospital, Inga Marie Nilssons gata 47, Malmö, 205 02 Sweden

**Keywords:** Overdose prevention education and take-home naloxone, Opioid overdose, Substance use disorder, Opioid overdose, Heroin dependence, Opioid use disorder, Addiction medicine, Qualitative research

## Abstract

**Background:**

People with illicit opioid use have a high risk of overdose related morbidity and mortality. A small group of *Supersavers* (i.e., individuals who have administered naloxone on three or more overdose occasions) carries a substantial burden by managing a high proportion of overdose reversals. Despite their critical role, little is known about how Supersavers’ experiences of repeated overdose reversals, their own overdose risks and its mitigation, or the support needed to sustain their engagement. This study examines Supersavers’ contextual experiences, their views on risks, responsibility, and overdose engagement, and their need for healthcare support.

**Methods:**

Semi-structured interviews were conducted with thirteen Supersavers recruited from Malmö needle and syringe program. Thematic analysis was conducted whereupon analysis was guided by Bourdieu’s theories on habitus, capital and field and Rhode’s ‘risk environment’ framework.

**Results:**

Supersavers were deeply committed to helping others and widely recognized for their skills, often having managed multiple overdoses prior to naloxone availability. This responsibility functioned as an embodied readiness to act, reflecting a habitus shaped through repeated exposure and supported by experiential (“street”) capital, including technical skills and peer trust. Access to training and naloxone strengthened this capacity, increasing perceived safety and reducing the physical and emotional burden of overdose reversals. While repeated exposure to overdose contributed to normalization, it also involved cumulative emotional strain, and most participants had not sought professional support despite experiencing multiple traumas.

**Conclusion:**

These findings show how Supersavers’ practices emerge through the interplay of habitus, capital, and risk environment, where engagement is sustained by responsibility yet shaped by structural constraints. Strengthening harm reduction responses requires not only continued access to naloxone and training, but also accessible, context-sensitive healthcare support that addresses both Supersavers’ own overdose risk and the emotional burden associated with repeated reversals.

**Trial registration:**

NCT03570099, registered on 26 June 2018.

**Supplementary Information:**

The online version contains supplementary material available at 10.1186/s12954-026-01487-x.

## Introduction

Among people who use drugs (PWUD), opioid related overdose is the worldwide leading cause of premature deaths [1]. Research has shown that a majority (72–74%) of individuals using heroin previously had experienced one or several clinically well-defined overdoses [2, 3]. A recent systematic review and meta-analysis estimated 2–6% of overdoses to be fatal among people who inject drugs (PWID) in United States, Canada, United Kingdom and Australia [4], indicating a high prevalence of direct or indirect morbidity symptoms among survivors [5]. Non-fatal overdose is thought to be one of the most prevalent risk factors when it comes to subsequent fatal overdoses [6, 7]. In many cases, opioid overdoses are preventable as these frequently occur in the presence of others [8–10]. If administered timely, the opioid antagonist naloxone is highly effective, its efficacy is well documented, and adverse effects are rare [11, 12]. In 2014, The World Health Organization recommended naloxone to be made available for all individuals at risk of witnessing overdoses [13]. Availability of take-home naloxone (THN) has increased in Europe, largely due to implementation of broad-scale regional or national programs, such as in Scotland and Norway [14, 15]. In most parts of the world access remains patchy and insufficient due to reasons such as stigma [16], legal barriers [17], and lack of resources, despite large-scale THN programs reducing overdose deaths on a population level [14, 18, 19], and have proven to be cost-effective [20, 21].

The responsibility that follows improved opportunities to save lives with naloxone is complex as it mainly relies on members of society with a longstanding history of stigmatization from the same healthcare system now depends on their commitment [22–25]. Previous studies have shown that responding to overdose events is associated with increased agency, pride, empowerment [26–28], as well as a strong sense of communal responsibility among responders [29–32]. At the same time, such situations often expose responders to intense stress and potentially traumatic events [32, 33]. Additionally, many responders operate in context marked by poverty, housing instability, criminalization and repeated exposure to overdose, which together shape how they experience and respond to these situations. Frequent involvement in overdose reversals, often without access to adequate support or time for recovery can lead to cumulative stress, burnout, and in some cases, avoidance or demoralization [34]. The strong social ties and norms of mutual care within these communities may generate a sense of obligation to continuously engage and intervene despite harmful consequences [31], while access to appropriate support for laypersons and harm reduction workers remains limited and insufficient [34–37].

A core subgroup of participants in opioid education and naloxone distribution programs (OEND) who repeatedly report using naloxone for overdose reversals has previously been described in the literature [38–40]. The term Supersavers was first used in a Norwegian context to describe individuals who had administered naloxone on three or more occasions [39] and subsequently been applied in a Swedish context [41]. Both Scandinavian studies found that a relatively small group of individuals accounted for a substantial proportion of overdose reversals.

Norwegian Supersavers were significantly younger than participants reporting zero to two naloxone-administered reversals, a pattern not observed in the Swedish cohort. Neither study identified significant gender differences, although Swedish sample included a higher proportion of women among Supersavers. Norwegian Supersavers more often reported current opioid use, whereas Swedish Supersavers were more likely to have received training and multiple naloxone refills through needle and syringe programs, suggesting more sustained engagement in injecting drug use [39, 41].

However, important gaps remain. Little is known about these individuals’ lived experiences or the long-term implications of repeated overdose response. Greater insight into high-frequency THN users, such as Supersavers, is needed to better understand this complex context. And to inform program design for more effective and sustainable engagement of those most likely to perform overdose reversals. When adequately supported with appropriate resources and training, OEND programs may help to reduce the gap between PWUD and the formal healthcare system; however, without such support, there’s risk of reinforcing that gap [42].

This study explores Supersavers’ experiences of overdose reversal and their engagement in OEND, with particular attention to their perceptions of personal risk, experiences of overdose, and access to support. The study is informed by ’the risk environment framework’ [43, 44] and Bourdieu’s concepts of *habitus*, capital and field [45, 46], which together provide a framework for understanding how overdose response practices are shaped by both individual experience and structural conditions.

### Theoretical framework

This study draws on Bourdieu’s concepts of on *habitus*, capital and field [45, 46], alongside the ‘risk environment’ framework [43, 44, 47, 48]. The risk environment framework conceptualizes drug-related harms as produced through interactions between economic, political, social and cultural structures, shifting attention from individual behavior to structural conditions shaping risk and harm [43].

Habitus refers to embodied dispositions shaped through social experience, capital to the resources that hold value within a given field, and field to the structured space in which these resources and practices gain meaning and are negotiated [45, 46]. Together, these concepts provide a framework for interpreting how overdose response practices are socially and structurally shaped.

Building on this framework, the study also draws on the concept of ‘street capital’, referring to knowledge, competence and skills valued within in the ‘street culture’ [49]. This form of capital may confer status within peer networks but is not necessarily transferrable to other social contexts.

In the analytical process, these concepts guided both coding and interpretation by directing attention to how Supersavers’ overdose response practices are socially shaped. Habitus is operationalized as participants’ embodied dispositions towards recognition and response to overdose situations, including norms around helping and risk-taking. Capital is examined in terms of experiential, social and practical resources, such as prior overdose experience, peer networks, and access to and overdose response skills and knowledge. Field refers to the local drug-use and harm reduction context in which these practices occur, including relations with other PWUD, access and availability of support services. These concepts were applied iteratively throughout the analysis to identify patterns across accounts and to inform the interpretation of emerging themes.

Together, these frameworks support an analysis of overdose response as socially embedded practice rather than individual action alone.

## Methods

### Aim

This study aims to examine Supersavers’ contextual experiences and views on responsibility and overdose engagement, their need for healthcare support and thoughts on whether these needs are adequately met by the healthcare system.

### Setting

Malmö, with a population of approximately 350,000, is the third-largest city in southern Sweden. It is located in Skåne County (population 1.4 million), approximately 30 min from Copenhagen, Denmark. Interview data were collected from Malmö Needle and syringe program (NSP). As part of the Infectious Diseases Department, it is the largest NSP unit in the region with over 500 registered adult patients in 2025. Malmö NSP has provided people who inject drugs with comprehensive healthcare services since the mid 80’s.

Swedish healthcare is tax financed and covered by the universal health insurance. Counties are self-governing in terms of providing healthcare for their citizens. A large-scale naloxone program was implemented in June 2018, in Skåne. At the time of the study, the program included more than 50 OEND-units [41] int the county, providing patients with training and naloxone. Procedures of training and distribution follows protocol, described in detail in Troberg et al., 2020 [50]. Included patients received their free-of-charge naloxone kit immediately after training. During the first seven years of the program, more than 3,350 at-risk individuals had been trained, and over 9,300 kits had been distributed (data collected in June 2025 from all units) in Skåne. Each kit contains two doses of intra-nasal sprays. Refills are provided for free at all units included in the program. Upon refill, patients are asked what happened with previous naloxone, whereupon counselling is offered if naloxone has been used for overdose reversal. Among participants returning for refill, over 1,500 reported having used their naloxone for overdose reversals.

At the time of participant inclusion, naloxone could be prescribed only to individuals at risk of opioid overdose. Naloxone was, and is continuously, distributed free-of-charge to all participants enrolled in OEND. However, for those not enrolled, naloxone could be prescribed by any physician or by nurses with a delegation. With prescription, naloxone is covered by medical high-cost protection, by which included medications are highly subsidized. Additionally, naloxone became available as an over-the-counter medication (prescription free) at pharmacies at the cost of approximately 50 USD (452–578 SEK) as of July 2024 [51].

### Design

#### Eligibility criteria and sampling

The study was advertised through posters at Malmö NSP. Participants were registered patients at Malmö NSP, enrolled in the THN program, recruited during their regular visit to the clinic. According to Swedish law, visitors must be 18 years or older and registered with their social security number.

Eligibility criteria included [1] having previously reported naloxone to be used for overdose reversal on three occasions or more [2] being able to speak Swedish. Inability to provide informed consent due to (Swedish) language difficulties, intoxication, or psychiatric disability was declared as exclusion criteria. Given that some degree of intoxication is common in this population, this criterion was applied pragmatically. If a potential participant appeared too intoxicated to provide informed consent, they were asked to return at another time. Capacity was assessed based on the individual’s ability to understand study information and engage in coherent conversation.

When returning for refill, patients were routinely asked about their use of previously dispensed naloxone. Clinic records identified 25 individuals who reported having administered naloxone on three or more occasions to reverse an overdose in another person. These individuals were considered eligible study participants. Frontline staff (registered nurses or assistant nurses) invited eligible patients to participate. A purposive sampling strategy was employed, whereby staff were asked to consider variation in gender and age, to the extent possible to capture a range of experiences across different demographic groups.

If willing to take part, patients were asked whether they preferred to be interviewed straight away, or leaving their number for scheduling a later interview. Prior to participants giving written consent, study participants received oral and written information about the study. Participants received a grocery store gift voucher valid for SEK 200 SEK (~ 20 USD) as remuneration, in recognition of their contribution. This was not presented as an incentive during recruitment.

Before submitting the application to the ethical committee, the interview guide was discussed and reviewed peer-representatives with lived experience. The study was performed in accordance with the Helsinki declaration and was approved by Regional Ethics Board, Lund (d.no. 2018/300 and d.no.2020–05176).

#### Sample

Eight of the 13 participants identified as male, on average 43.5 years old (range 30–56). All but one participant had managed at least one overdose prior to OEND implementation. A majority (*n* = 10) had enrolled and received their training with an initial naloxone kit at Malmö NEP. Prior to the interview, records showed that Supersavers had reported a mean number of 5.5 overdose reversals with naloxone, in total 71 (range 3–14). However, when asked whether these records were correct, all except one participant reported occasions were representative of the total number of reversals in which they had used naloxone. Except for one participant, all reported having engaged in a greater number of overdose situations in which they had administered naloxone, adding up to a total number of 131 (mean 10.1; range 3–30) [Additional file 1].

#### Data collection

The interviews were conducted in a secluded area at Malmö NSP between December 2022 and April 2023. Before the interview, participants were presented a list of dates for when they had returned to Malmö NEP for refills and reported naloxone used for overdose reversal. They were asked if this list correctly represented the (amount of) overdose reversals they had managed or if they wanted to alter the number. Thirteen face-to-face interviews were conducted, eleven were conducted by both authors, P.I. and K.T., while the last two were conducted by P.I. alone. Interview times ranged from 12 to 58 min, with a mean of 31 min. Semi-structured interviews with open-ended questions were used in an explorative design aiming to capture Supersavers lived experiences and perspectives on repeatedly engaging in overdose management, overdose risk awareness, access to and carrying naloxone and whether their engagement in OEND had changed their perspectives on themselves and their behaviors. Questions also concerned availability of acceptable support and whether it was sufficient in relation to their needs.

At any point during the interview, participants could question or comment on their interpretation of the questions, or their answers thereof. Participants were also asked if there was anything else they would like to comment on or if the questions hadn’t been aligned with things they thought were important, either to themselves or their peers.

#### Researcher characteristics and reflexivity

Both authors have extensive professional experience working with people who use opioids, including more than 15 years in clinical and support roles related to heroin use and injection drug use. Author KT is a registered nurse and holds a Master’s degree in sociology and a PhD in Medical Science. PI is a counsellor and has longstanding experience of being involved in research within the field. Both have been involved in harm reduction initiatives, including the implementation of a naloxone program over the past eight years, with a focus on overdose prevention and education for patients, staff, and potential bystanders.

While the authors have prior experience working clinically with this population, they were not in direct care roles or responsible for participants’ treatment at the time of data collection. This was considered important in reducing potential power imbalances and facilitating open dialogue during interviews.

The authors’ professional backgrounds and commitment to harm reduction informed both data production and analysis, particularly in recognizing and valuing participants’ lay expertise in overdose response. At the same time, reflexive discussions were undertaken throughout the analytic process to consider how prior assumptions and experiences may have shaped interpretations of the data.

As noted by Anna De Fina, interview narratives may involve elements of self-presentation. In this study, however, the researchers’ longstanding involvement in the harm reduction setting and familiarity with participants may have facilitated more open accounts, although the possibility that participants emphasized responsibility and competence cannot be excluded [52].

#### Data analysis

The interviews were recorded with a digital audio recorder and transcribed verbatim by a research assistant. Applying thematic analysis, as described by Braun & Clarke (2006), interviews were initially listened to and read repeatedly by both authors separately, securing transcript accuracy and familiarizing with the material, achieving a sense of the data as an entirety and identifying patterns and ideas [53, 54]. Notes taken during the interviews formed an additional layer of information. Initial codes were created and assigned. Segments of text were labelled with keywords which summarized the essence of experiences and thoughts whereupon codes were assembled into overarching categories, i.e. themes. Codes and themes were then reviewed and refined to assure the significance of the data in relation to the theme and the full dataset, prior to deciding on final definition and naming of the themes and creating a thematic analysis report. The analysis combined inductive and deductive approaches. An initial inductive, “low interference” analytic strategy was applied, aiming to produce a close and straightforward account of Supersavers’ lived experiences [55]. Interview transcripts were coded with minimal theoretical pre-structuring, and themes were developed based on recurring patterns in the data. Quotes representative of each theme was presented with identifiers for participant number, gender, and age span to preserve anonymity.

Subsequently, during the interpretive phase, the analysis was informed by the risk environment framework and Bourdieu’s concept of habitus, capital and field. These concepts were operationalized as follows; habitus was examined through participants’ embodied dispositions; capital was conceptualized in terms of skills, experience, competence, i.e. both practical and social resources; and field referred to the local harm reduction and drug-use context in which these practices were embedded. The risk environment framework further guided attention to the social and structural conditions shaping overdose events and engagement thereof. This deductive interpretation was used to explore the relationships between codes and themes, whereupon four central themes developed; Supersavers’ (1) Experiences and roles within the context of drug-use; (2) Overdose management practices and risk awareness; (3) Responsibility and consequences of overdose engagement; (4) Traumas and support.

## Results

### Experiences and roles within the context of drug-use

When participants were asked how they had come to be involved in multiple overdose events, most reported that they were either individuals whom others routinely contacted for assistance during an overdose or that they were already present at the location when the incident occurred. A smaller number attributed their presence to activities related to buying or selling drugs, whereas others explained that they were present in their capacity as a partner, acquaintance, or friend of the person experiencing the overdose. In a few cases, participants described their involvement as purely coincidental.

Supersavers generally described themselves as being someone who others trusted, whom others feel they could turn to. They claimed having been “in the game” for a long time, being well-known for their skills and for having dealt with multiple overdose incidences, long before naloxon became available. Most portrayed themselves as being part of what they termed as “*the old school*”, having a strong moral compass, with shared common values.

*“We belong to a group of older and experienced individuals who unfortunately lost many. Those who’ve survived and still are around know that I’ve helped and revived a lot of people in our circle of friends. They know me and know that I have naloxone.”* [Man, late 40s].

Most described overdose management in terms such as “*like riding a bike*” feeling calm in stressful situations, relying on their skills, knowledge and experience. Some, however, expressed continuous apprehension, worrying what would happen if the naloxone wouldn’t have the desired effect. Despite ample experience, a man in his mid-fifties revealed still feeling anxious every time he was called upon for help, even though having successfully reversed more than 30 overdoses by administering naloxone. Although continuing to worry, he felt grateful when given the opportunity to help others as he truly felt that this was his calling. He expressed an unwavering willingness to continue responding to such calls, provided that his efforts were not taken for granted and that he did not feel exploited.

While most participants found it difficult to appreciate if training and access to naloxone had changed their perceptions of themselves, to some, the impact OEND had made was quite remarkable:

“*It has changed my life*,* bringing more balance into life. I’ve become wiser in my ways of living. It has definitely made me a different man.*” [Man, mid-50s].

Others denied that access to naloxone had changed their view of themselves, claiming they had simply continued doing what they always had done, helping others in need, being someone others trust and turning to for help when in need of assistance.

### Overdose management practices and risk awareness

Throughout the interviews, Supersavers described several ways by which training and access to naloxone had not only made overdose reversals a lot easier than before as they didn’t have to work as hard. Carrying, or having naloxone where drugs were used, and being able to modify their techniques had made them feel safer. Although recommendations of safe use were not always applicable, they claimed prevention training having made them more aware. Participants emphasized more routinely considering options before injecting alone or in situation or space which was regarded to be unsafe.

#### Switching techniques

All agreed that hands-on training and access to naloxone had been a gamechanger, with many participants expressing that minutes used on training were “*the best spent minutes in your lives*” [man, late 40s]. It had made them feel safer, making overdose reversals easier and less exhausting. Having been in the game for a long time, they reckoned that it was their way of doing things that had gotten them this far. Although having been somewhat successful when applying old techniques, such as injecting a saline solution or large quantities of amphetamine, often accompanied by cold shower and subjecting the victim to excessive pain, these methods were regarded as messy, inefficient, and required a lot of work. Since enrolling in OEND, all denied using old techniques, mainly due to the efficiency of the new. Participants sought for naloxone to always be available, as shared by one man:

“*If there’s a bunch of people and a lot of junk [heroin] and other drugs in the flat*,* I always say “so*,* in this bag I have naloxone if anyone needs it”*,* or I’ll just slam it right at the middle of the table*.” [Man, early 30s].

Participants were commonly announcing where naloxone was kept, in many cases for their own safety. If naloxone wasn’t carried, it was kept where drugs were normally used, to be available when needed. Several participants shared how they had placed naloxone at strategic locations or announced where to find it, by putting up posters or making sure that staff at low threshold facilities had access.

Participants denied that previous experience of anger and irritability from overdose victims would stop them from getting involved in a future overdose situation. The majority stated that the anger exhibited by victims after reversal was a recurring phenomenon which they were used to. Quite a few mentioned own experiences of confusion, anger and stress from being brought back from a near-death experience by the use of naloxone:

“*Many fear ruining someone else’s high as people may get irritated when they wake up. They are simply unaware of what has happened. And I myself have experienced exactly that. I’ve overdosed and then I woke up and was really pissed off. But at the time*,* I just didn’t realize that I was almost dead… so… they can get as angry as they want*…” [Woman, early 30s].

Own experience of overdose had given the perspective and understanding of what it could be like for others in similar situations, seemingly making participants more resilient to the anger of others.

#### Calling emergency services

Except for two participants, all immediately claimed they wouldn’t hesitate to call an ambulance if needed. Encounters with ambulance staff were generally described in positive terms. They were reportedly treating Supersavers with respect, often thanking them for their help:

*“They were really happy*,* because the staff*,* the ambulance staff*,* yeah*,* they hugged me.”* [Man, mid-50s].

Turning to the two participants claiming they would probably not call an ambulance, the woman in her mid-fifties maintained never to have called an ambulance, despite having reported over 20 reversals involving naloxone. She simply did not own a phone. In most situations, she had been alone with the victim when an overdose occurred, whereupon she had to handle it by herself not wanting to leave the victim. A man in his early thirties, initially stated that he wouldn’t call paramedics as the victim was likely to refuse transportation anyway and would probably also get angry if he called. Though, further into the conversation, he changed his statement saying that it all depended on the situation and on who the victim was. He paused, then added that in most cases, there was no need to call, as it would be a waste of public resources. He strongly denied that his reluctance had anything to do with fear of police involvement or other possible negative consequences, explaining that from his own experience and contrary to what others believed to be true, police weren’t interested in arresting people for illegal possession of drugs if there was a life-or-death situation as their main priority is to save lives:

*“When it comes to the police*,* they want to frame all of us who use heroin as it gives long-term prison sentences. But*,* when people are dying*,* they don’t give a shit about drugs. People still don’t get that. I’ve never been in an overdose situation where a police officer suddenly goes “Oh*,* I wanna check your pockets”. [In those situations] they don’t give a fuck about the drugs.”* [Man, early 30s].

Other respondents made similar statements and generally, Supersavers did not perceive police involvement as a barrier for calling the emergency services (EMS). Quite a few participants reckoned there had been a change over the years, as the police didn’t accompany EMS as frequently as before. Lately, ambulance staff were considered to be helpful and to behave respectfully towards respondents, which was a change in relation to past experiences.

Although Supersavers did not personally fear police involvement, they reported that individuals who sought their help frequently perceived such involvement as a significant barrier to contacting the EMS. One participant, a man in his late forties, recounted an incident in which a woman called him in desperation as her boyfriend had overdosed in a public restroom at a popular amusement park and was already exhibiting signs of hypoxia. When he told her to call an ambulance immediately, she responded that she couldn’t as she was in possession of a substantial quantity of drugs.

“*She was going to leave him there*,* taking the drugs with her*,* but I said*,* “you are not leaving him. If you do*,* I will tell everyone what you did.” I threatened her and said: “You will stay put and perform rescue breathing until I get there with the naloxone*,* or else I will turn you in”. She stayed and did what she was told as she was afraid that I would report her to the police. I managed to bring him back by giving him naloxone*,* but it was only because she had continued rescue-breathing. If she’d left him*,* he would have been dead.*” [Man, late 40s].

He rapidly added that of course he would never have told the police. He wasn’t a snitch, he simply had to make the person realize how serious the situation was.

#### Safety recommendations

Most described having become somewhat more cautious than before when it came to their own drug use, employing strategies such as injecting slowly, starting off with a smaller dose when uncertain about substance’s quality or potency. However, they also described significant challenges in adhering to recommendations on safe use. Avoiding solitary use was for instance not always possible. In many cases, using alone was perceived as a safer alternative, particularly in relation to concerns about the presence of others whom they did not trust. Furthermore, participants frequently described how acute withdrawal and intense craving created a sense of urgency that made it difficult to follow safer-use recommendations, as the need to inject quickly often outweighed considerations of safety.

“*When you’ve decided to get that fix*,* then you don’t care how. You just have to do it. It doesn’t matter if you’re alone somewhere or in the company of ten others. That drug is a real bitch when it gets a hold on you.*” [Man, mid-40s].

Participants also described that accidently “going over” sort of came with the territory of being addicted to heroin. For some, “going over” was hard to avoid when wanting to get as close to death as possible.

“*It’s hard [taking precautions]*,* as many say*,* the best kick is the one closest to death. That state is what many try to reach and for most*,* heroin alone is not enough. To get that high from heroin you need really high doses. And moneywise*,* a gram a day will not be enough*,* so you have to pop pills to feel more*,* and that’s when it gets dangerous. When you mix with pills*,* such as benzos and Lyrica [pregabalin]*,* that’s when things start to happen*,* and that’s when things become lethal*.” [Man, early 40s].

Heroin in itself not being dangerous was persistently explained by a few male participants. They expressed the belief that fatal overdose was unlikely to occur from heroin use alone and that the significant risk emerged primarily when combined with other sedatives. Concurrent use of heroin, alcohol and benzodiazepines was frequently reported, mostly due to financial constraints, limiting access to sufficient quantities of heroin.

#### Own risk awareness—us and them

Generally, there was a clear distinction between male and female participant responses when asked to describe their perception of own overdose risk. All female participants, except for one not using heroin or other opioids, immediately responded that they themselves were susceptible to overdose and that it would be stupid denying it could happen to them. In contrast, only two male participants explicitly described perceiving themselves at risk of overdose, while the majority positioned themselves as “not like others”, emphasizing increased control, experience, and tolerance over time. These narratives reflect a broader tendency to construct distinction from other drug users through competence and self-control. Although these accounts were consistent across participants, they were articulated through different justifications and self-descriptions.

“*Somehow*,* I have realized that I’m not as fucked up as others. I have a certain control over it. I’m a drug addict*,* but it’s not that I’ve got that urge to be close to death. I believe I understand myself a bit better.*” [Man, mid-40s].

“*You know*,* I’m different from the others [heroin users] as many do drugs to get as wasted as possible*,* I only do drugs to get as much pain relief as possible. That’s where we differ.*” [Man, late 40s].

These accounts illustrate a common positioning of self as controlled and knowledgeable, often contrasted against an imagined “other” characterized by loss of control or excess use. This framing reinforces perceived separation from overdose risk despite long-term opioid use. To them, these traits, experiences and skills had gotten them this far, made them into “survivors” and separated them from “others”. “Being greedy” or “keen”, were common flaws used to describe novices or those referred to as “fucking junkie tourists”, having no idea of what they were dealing with:

“*They buy their heroin and then they’ll go back to wherever the fuck they’re from. It is dangerous. With no experienced users around and being too keen …”* [Man, mid-40s].

Inexperienced users were often mentioned as they often caused an array of problems. One woman in her early thirties with a long experience, rich in street capital, as she had used heroin for more than half her life, described how novices caused a lot of stress when they withheld vital information from the heroin dealer expertise.

“*I guess*,* I’m used to heroin*,* they weren’t. So*,* they popped some benzos without my knowledge*,* and I had asked them about benzos before*,* because to me*,* it’s vital information. Because if that’s the case*,* it [the heroin] will hit them harder. I need to know. How will I otherwise be able to… otherwise it will be hard for me to give them the right amount. They said*,* “no no*,* no benzos*,* no benzos”. So*,* I just*,* ok. So*,* the first one took a hit*,* and it didn’t take long before she was unconscious.*” [Woman, early 30s].

She continued describing how both were given naloxone, but only one of them responded whereupon the ambulance services had to be called, causing an enormous amount of stress as it happened at her apartment. Their behavior was deemed reckless and destroyed the atmosphere and her own anticipated high.

Incidents where Supersavers had to call EMS and “babysit” due to novices’ greed and recklessness were frequently reported. However, situations when similar behavior was exhibited among more experienced peers, annoyed Supersavers even more as they felt responsibility were forced upon them by peers who should have known better.

*I’m so fucking tired of all those junkies and their fucking pill popping. So no*,* I get angry. It’s so fucking convenient*,* if you are going to take an overdose – go somewhere else. Don’t hang around here*,* because then you’ll leave us with responsibility. That’s such a nasty thing to do.*” [Woman, mid-50s].

Despite this selfish and careless behavior which abused friendship and trust, they all claimed they would do whatever it took to save their lives. However, as these individuals were not very popular to have around, and would be avoided if given the option, this behavior could leave them in a more vulnerable position of overdose death.

### Responsibility and consequences of overdose engagement

#### Caring for the wellbeing of others

Participants expressed intense anger when confronted with situations in which responsibility for another’s care was imposed on them. Frequently, this anger arose from the fear of losing yet another person close to them mixed with the perception that the victim did not grasp the severity of the situation. In addition to being left with the responsibility of friends of family members, or sometimes acquaintances, one woman described a situation in which she had summoned the EMS. Once revived, the friend refused transport. When the ambulance staff left, they also left her with sole responsibility. She described the feeling of being trapped. She said that this experience engendered profound resentment and led her to adopt an increasingly indifferent attitude towards other overdose-situations.

Although anger commonly surfaced when someone with whom participants were emotionally invested in failed to recognize how close they had come to death, the core of their accounts was deep-rooted care for the wellbeing of others. They bore a profound sense of responsibility towards others, as described by a female participant:

“*I feel that we have a responsibility towards each other*,* when we are in each other’s company and we use together*,* and yes*,* of course it’s annoying when someone overdoses. But I would never leave someone behind. Even if I don’t have naloxone*,* then I would call an ambulance*,* but I would never just leave.*” [Woman, early 30s].

Another woman described finding a young man overdosing, not far from a children’s daycare center where children were playing. Realizing that the actions which she would have to take could worry or scare the children, she informed one of the staff minding the children. The kids were ushered off, before she tended to the young man. She managed to revive him and escorted him to his home, there finding his drunk parents. As she left, she urged them to keep an eye on him.

*“I haven’t seen him since then*,* but it was very uncomforting*,* him lying in the bushes*,* it was such a stark contrast to the kids playing on the other side… and him almost dead there in the bushes. And he was so young*,* only 19.”* [Woman, late 40s].

Having successfully revived this young stranger she was left feeling grateful, yet apprehensive. Despite overdoses generally being depicted as not such a “big deal” but rather being a “*normal part of everyday life*”, the more participants talked about their experiences and how it made them feel, the more they described reliving traumas, sadness and anger. In order to be able to carry on, these feelings had to be ignored.

#### Access to naloxone is a blessing

Although many described situations in which their engagement in overdose reversals had caused them distress and bereavement, overdose engagement was mainly described in positive terms, as a blessing. To most participants, access to training and naloxone was experienced as empowering and as an opportunity to do good, fostering feelings of pride and increased safety. Naloxone was described as a blessing and a gamechanger in everyday life, and the responsibility associated with it was generally not perceived as burdensome. While this overall framing was shared across participants, it was expressed with slight variation in emphasis and personal meaning.

*“…not a huge thing*,* not like I’m carrying a heavy responsibility. I see it more like being a fellow man in a world which includes a limited number of people.”* [Man, early 30s].

*”Sometimes*,* when I feel low*,* then at least I can feel that I’ve done something good for someone else [by engaging in overdose reversals].*” [Woman, early 30s].

Generally, access to naloxone had not fundamentally altered their sense of responsibility, as the majority had responded to multiple overdoses prior to its availability. Rather, naloxone was described as making these situations feel safer and more manageable, reinforcing its role as a supportive rather than burdensome intervention.

Although he initially stated that engaging in overdose reversal “certainly empowers you” and made him feel proud, one male participant went on to explain that overdose reversals had become neither unique nor noteworthy to him, being part of normality due to their frequency, stating:

“*What I believe is… that if you live this life*,* these are the consequences you have to deal with. It comes with the package of doing drugs*.” [Man, early 40s].

Participants’ overdose response was intertwined with their everyday lives, leaving little room to step back or imagine an alternative path. For many, the chance of “getting out” seemed limited. Instead, they felt compelled to carry on, guided by a sense of responsibility to help others, even when doing so took a toll on their own well-being.

### Traumas and support

#### Unprocessed trauma and emotional pain

While none of the participants reported having experienced negative consequences in relation to overdose reversal engagement since enrolling in OEND, all described multiple experiences of traumatic events which had a major impact on their general wellbeing throughout life. Traumas were recognized for making them well equipped and committed as Supersavers however also as adding to anxiety and fear of failure. These experiences, such as having lost friends and family members to overdose and suicide, had mainly been dealt with and shared by others within their small network of trusted individuals, predominantly described among female participants.

“*[Overdose] is really traumatic*,* it’s horrible*,* but no*,* I’ll deal with it with my friends.*” [Woman, mid-50s].

Only three participants reported having sought professional support to deal with previous trauma, yet most described an unmet need for accessible and acceptable services. Those who had accessed support did so through their NSP or OAT clinic, where they felt they received non-judgmental assistance.

Issues with trust were aggravated by having lost several friends and family members over the years. For some participants, the shrinking network of trusted peers made it harder finding someone to turn to. This pattern was especially apparent among male participants, who described seldom seeking professional support and having only a limited number of trusted individuals within their close network they could confide in. In response to questions about whom they might seek support from during a crisis, or if someone they had tried to revive were to die, one young man offered a straightforward reply:

“*I don’t know. I have never turned to anyone for support when someone died. So*,* probably no one.*” [Man, early 30s].

One participant described how repeated loss and grief had become visible as he and his brother had made a long list of names of relatives and close friends lost to overdose over the years. Since then, he had also lost his brother, a trauma he still carried with him.

“*This thing with my brother*,* I realize that I have to deal with it at some point*,* it’s the greatest trauma of my life.*” [Man, mid-40s].

Not only did all the trauma and grief put him in a vulnerable situation, but having lost those closest to him also made it increasingly harder for him to find someone to confide in. Another participant described struggling with unbearable grief and loneliness, after losing his brother to suicide during his adolescence.

“*No one offered me counselling when my brother killed himself. I had no one to turn to.”* [Man, early 40s].

Despite the severity of the traumatic experience, he was not offered any form of professional support. Now in his early forties, he recognized the substantial and enduring impact the event had exerted on his life, acknowledging it as an issue that required attention. Other participants described a tendency to distance themselves from the internalization of trauma by normalizing or rationalizing comparable experiences. Most reported feeling no acute need to seek help and expressed resignation about “where to begin,” emphasizing that more immediate concerns consistently took precedence. Illustrating his coping strategy, one male participant in his early thirties remarked, “*The trick is to have so many traumas that none of them stand out*.”

None of the participants reported having failed to reverse an overdose since gaining access to naloxone. However, all but one had prior experience of overdose situations before naloxone was available, including instances where resuscitation attempts were unsuccessful. Several participants described how these earlier experiences remained vivid, particularly when encountering similar situations more recently and how they had to manage associated feelings of panic and anxiety. When asked how they might have responded emotionally to an unsuccessful reversal, some expressed concern that such an outcome could have a significant impact on their well-being. However, most described these potential experiences in a context of already accumulated trauma, suggesting that it would be just another trauma which would be “another” difficult experience added to all the others.

#### Naloxone program support and vulnerable groups

When asked what the OEND program should offer to ensure sustainability and mitigate emotional stress from overdose engagement, most participants did not express any needs of their own, though stressed the importance to focus on the needs of those engaging in overdose reversal for the first time. They suggested there should be detailed and clear information on where patients could seek support, and to repeatedly remind patients of the possibilities of easily available and accessible support. They also stressed flexibility around appointments, as keeping track of these could be a challenge, or even a barrier to some. Furthermore, they also highlighted that for some, the need for counselling was not always imminent in direct connection to an incident, thus the need could become apparent later.

All participants emphasized that naloxone should be made more available and accessible. A few participants also highlighted that there were major differences in availability and accessibility depending on where in Sweden you live. Allowing naloxone to be made available in public places and equipping police, security personnel and low-threshold staff with naloxone was suggested as low-hanging fruit, if the goal was to make naloxone available where and when overdoses occur.

*“I think security guards and police should carry it. It should be a lot easier to access it than having to turn to NSP or OAT. One should be able pick it up for free at pharmacies.”* [Man, early 30s].

Due to different reasons, such as stigma and fear, certain populations were portrayed as being more vulnerable, being “out of reach”, wanting to stay “under the radar”. Concerns were shared especially about young people using tramadol, perceiving pills “not to be dirty”, as heroin, but as a medication and being less aware of the dangers. Undocumented young people were thought of as an even more exposed group.

*“There’s a huge group of heroin users here in Malmö crazy-scared of authorities generally. They are super destructive when it comes to drug use /…/ They’re too scared to talk to anyone. They don’t even dare to do the smallest of things like buying chocolate. They are really paranoid about everything and yeah*,* if the cops get them*,* they’ll likely get deported for being here illegally.*” [Man, mid-40s].

At the time of the interviews, naloxone was available only for individuals at risk of overdose which left those with access with an inherent responsibility to provide people not reached by the program with naloxone and information. Perceiving this deficiency in harm reduction outreach they developed unsanctioned underground networks. In addition to serving a healthcare system, by which many had experienced stigma and being negatively judged, Supersavers provided a connection between that very healthcare system and individuals who chose to stay under the radar.

As portrayed by most participants, they simply distributed information and naloxone to those not in possession, increasing awareness and availability in their community. One man expressed his engagement in underground distribution with a big smile explaining his view:

*“We distribute to staff over there [low threshold housing] as they can’t acquire naloxone. And we are happy to do so*,* everything else would be crazy. I think it’s really amazing. This is the way it should be done; you’ve involved us knowing that we don’t think about the law like you.”* [Man, early 40s].

Participant engagement filled an important gap between healthcare and individuals within their community who, for different reasons, found it hard to reach healthcare services, including overdose education and THN. Although participants described feeling exhausted and trapped by their repeated involvement in overdoses, access to naloxone made these situations easier to manage and increased their sense of safety. It reduced the chaos associated with responding and increased their feeling of empowerment.

## Analysis and discussion

This study identified a core group of highly skilled and trusted individuals repeatedly engaging in overdose reversals, with a strong feeling of commitment and pride. Supersaver’s testimonies showed a large proportion of underreporting in use of naloxone for overdose reversals. All except for one participant had engaged in more overdose situations than previously accounted for and had additionally engaged in multiple overdose reversals prior to naloxone becoming available.

The definition of “Supersaver” warrants a brief consideration. While we applied a “three or more” reversal threshold in line with prior Scandinavian studies, our findings suggest that the responding to overdose situations by administering naloxone is a more common than what is reported by participants. Presumably, fixed thresholds may not fully capture the extent of this activity, particularly as naloxone programs expand over time.

Four central themes emerged; Supersavers’ (1) Experiences and roles within the context of drug-use; (2) Overdose management practices and risk awareness; (3) Responsibility and consequences of overdose engagement; (4) Traumas and support. Findings were interpreted by employing Bourdieu’s theories on *habitus*, capital and field and Rhode’s framework on structural vulnerability, allowing for an analysis which shows how conditions of everyday life are shaped by the structuring structures.

Drawing on Bourdieu’s theory of habitus, participants appeared to share a set of lived conditions and embodied dispositions shaped by their experiences within the street field. Their collective habitus [46] encompassed tacit rules, shared assumptions and deeply rooted beliefs (doxa) which guided behavior and perception within this social field [45, 46]. As such, their authority stemmed not from institutional recognition, but from lived credibility and the accumulation of social capital within the street field.

Despite facing significant stigmatization and marginalization from mainstream society, many participants described themselves as experts within their field. Their integrity was often seen as something earned through longevity and survival in “the game,” and resonated with Ilan’s [56] concept of a “*culturally mediated notion of dignity*” (56: p. 3) a moral code rooted in shared street values and lived experience. Their perceived expertise was closely tied to their intimate knowledge of drug use, overdose response, and harm reduction practices. For the majority, it was also closely linked to own overdose history. These competencies, along with being regarded as trustworthy and capable, somewhat distinguished them from the descriptions of those seeking their help. Experiences which also encompassed positive encounters with ambulance staff and police officers during previous overdose situations. As opposed to those seeking their help, Supersavers all denied fear of negative consequences of having to call EMS, which, to the best of our knowledge, has not previously been described in literature.

Problematization in regard to the structural vulnerabilities within the ‘risk environment’ faced by PWUD when trying to adopt recommendations in ‘real life situations’ has previously been described in international research [57–60]. These challenges were also apparent among our participants. Data from the two largest Swedish naloxone programs demonstrate “the logic of practice” [46] when calling EMS, which was reported in nearly half of all overdose situations where naloxone had been administered [40, 61]. Though, when compared to international data, reasons for refraining from calling varied [62, 63]. Whereas 60% of participants enrolled in OEND in Skåne, Sweden, refrained from calling as they did not find it necessary, 16%, respectively 4%, refrained from calling due to fear of police or social services involvement [40]. This suggests a generally low fear of negative consequences when calling, compared to national and international data [64–67]. While Supersavers themselves reported little concern about potential repercussions when calling EMS, they frequently described instances where peers reached out to them for assistance instead of contacting EMS directly which suggests structural vulnerabilities within the ‘risk environment’ impacting different groups in different ways, forcing some individuals into less favorable behaviors, in relation to health and safety, due to fear of police involvement and lack of options. Previous findings have shown calling EMS being translated into calling the police [65], thus, fear of punishment and incarceration due to police involvement has traditionally been the most frequently reported reasons when refraining from calling an ambulance [38, 66, 68–70]. These findings suggest that individuals with less experience may face a heightened risk of drug-related morbidity and mortality due to their structural position within a stigmatized and criminalized environment. In this context, criminalization may actively contribute to harm by creating barriers to timely medical care in overdose situations.

In addition to having extensive experience of overdose management both before and after naloxon becoming available, abandoning old methods for naloxone and applying new techniques led to reversals becoming less “messy” and more efficient, implying a reduced need for contacting EMS, despite being strongly emphasized during training. Similar to previous international findings expressed by both peers and peer workers, participants reported feeling confident both with overdose management and of knowing when to call, though felt it would be a waste of resources to always call [66, 71], and that calling could raise unnecessary and unwanted attention [58].

Supersavers experiences of positive encounters with ambulance personnel and police officers seemed to differ from the collective habitus of those seeking their help. Previous experience had made Supersavers confident that police officers attending overdoses would not cause them any trouble, as saving lives were also the primary focus of the police. Positive experiences such as these could present an opportunity to bridge the gap between PWUD and the healthcare system, at least to some extent, though implying the need for targeted interventions, preferably led by trusted individuals with own experience [72]. Unfortunately, Swedish Supersavers currently lack the legal opportunity of engaging sanctioned peer-training and naloxone distribution, through which information and naloxone could reach individuals wanting to “stay under the radar”. Most Supersavers did, however, proudly declare their engagement in unsanctioned distribution of naloxone and information.

While using alone has previously been described as a safety precaution among female heroin users [57, 73], solitary use was also common among both male and female “Supersavers,” although the motivations and perceived safety benefits varied. Participants described avoiding inviting others into their homes in order to reduce “heat,” as the presence of others could increase the risk of eviction. For some, avoiding social contact during drug use was also a strategy to reduce exposure to violence, exploitation, or theft. However, limited access to trusted social networks further constrained opportunities for safer use, as maintaining stable and reliable relationships around drug use was often described as difficult or unsustainable. These findings align with previous research suggesting that solitary injecting may be shaped by concerns related to safety, stigma, and control [74]. As noted by Moore and colleagues, tensions between trust and exploitation may further undermine harm reduction advice such as avoiding solitary use [59].

The complexity of structural vulnerabilities and contextual circumstances often left Supersavers with very limited options, often due to desperation, fear, perpetuated stigma and shame. This facilitated isolation and forced them to hide their drug use, very similar to the situations previously described by international research [75–77]. Together, these findings illustrate a tension between knowledge and practice, where participants’ awareness of overdose risk and prevention strategies is mediated by structural vulnerability and constrained choices. Experience of overdose response may shape how risk is perceived and normalized, but the ability to act on this knowledge is limited by social, economic and environmental conditions. Practices, such as using alone, should not be understood simply as individual risk-taking, but as situated responses to a risk environment characterized by stigma, instability and limited resources.

There seemed to be a gendered discrepancy both in how participants perceived their own overdose risk and access to support. While male participants demonstrated extensive knowledge of overdose risks, they often positioned themselves as unlikely victims. In contrast, female participants more readily acknowledged their own vulnerability to overdose. Among male Supersavers, previous overdose experiences were typically attributed external factors, such as contaminated or unusually potent substances, whereas overdose among “others” were sometimes associated with greed, lack of skill or knowledge.

Previous research has shown that being older and more experienced is associated with a lower perceived risk of overdose [78]. Additionally, individuals tend to attribute their own overdose incidents to contextual factors while viewing others’ overdoses as a result of personal shortcomings, reflecting an actor-observer bias [79]. However, these explanations do not fully account for the gendered patterns observed among participants. From a Bourdieusian perspective, such differences can be understood in relation to gendered dispositions shaping how risk, competence and vulnerability are perceived and expressed. Within the drug-use field, forms of ‘street capital’ may be tied to demonstrations of control, experience and resilience [49]. In this context, downplaying personal overdose risk and emphasizing competence may function as a strategy for maintaining status and legitimacy, particularly among men. This aligns with research showing how drug use practices among men can be associated with masculinity, socialization and belonging [80].

These gendered dynamics should also be situated within a broader risk environment, where structural and social conditions shape both exposure to overdose and responses to it. Although specific mechanisms remain underexplored, Bardwell and colleagues suggest that gendered norms and practices may influence both risk-taking, and help-seeking behaviors [81]. Together, these findings indicate that gendered habitus may shape not only exposure to overdose risk, but also how individuals position themselves in relation to that risk and to others within the field.

An important finding is the relationship between repeated engagement in overdose response and participants’ perceptions of their own risk. Despite extensive knowledge of overdose recognition and response, this did not consistently translate into heightened perceptions of personal vulnerability. Instead, repeated exposure appeared to normalize overdose risk and foster dispositions of familiarity and control, consistent with Bourdieu’s concept of habitus. This aligns with Hanoa and colleagues, who describe how overdose risk may be downplayed or interpreted in complex ways among PWID [82].

These perceptions were closely linked to engagement in overdose response. Understandings of personal risk shaped how overdose situations were interpreted, as well as individuals’ sense of responsibility to intervene and their thresholds for action. In this way, risk perception and overdose response experience were mutually reinforced, with accumulated experiential knowledge both shaping and sustaining repeated engagement.

While none of the participants reported failed reversals following access to naloxone, most had prior experiences of unsuccessful resuscitation attempts, which remained emotionally significant and shaped subsequent responses. Participants framed potential future failure within a broader context of repeated trauma and loss. In a risk environment characterized by ongoing exposure to hardship, unsuccessful outcomes were often understood as part of an accumulation of experiences rather than exceptional events. This normalization may reduce the perceived need for formal support, while also obscuring the emotional burden associated with repeated overdose response.

Despite multiple reports of unresolved trauma among participants, consistent with the findings of Song and colleagues [32], support was primarily sought within trusted peer networks, while professional help was rarely accessed. Among Supersavers, only three participants, two of whom were female, had sought professional support. In crisis situations, all female participants reported having someone to turn to within their close network, whereas the majority of male participants indicated that they did not.

Masculinity norms have previously been suggested as a possible explanation for men’s resentment to seek help while suffering from depression and suicidality [83]. In relation to Bourdieu’s theories, dispositions shaped by long-term exposure to risk may normalize self-reliance and discourage expressions of vulnerability as it could be interpreted as a sign of weakness [46]. What may be described as masculinity norms may, from this perspective, be understood as part of a gendered habitus, where emotional control and independence are valued and taken for granted and part of the norm. The ability to “handle one’s own”, without seeking support, may function as a form of symbolic or “street” capital, contributing to recognition as experienced and resilient. Expressing a need for emotional support may risk undermining this position.

These practices are shaped by the risk environment as structural barriers, such as stigma and limited access to appropriate and acceptable services, may further discourage engagement with formal support systems, whereby reinforcing reliance on informal networks [81]. The absence of close support among the majority of male participants may therefore reflect not only individual reluctance but also accumulated social and structural vulnerabilities.

As a result, these findings point to a particular vulnerability among ageing male Supersavers, were limited social support and reluctance to seek help may increase risk of isolation and overdose death. While previous research has shown that women who use drugs often report lower levels of social support [84], the present findings suggest that gendered patterns of help-seeking and social connectedness may operate differently within this group. However, our previous findings show a reduction of overdose death in our region after implementing a broad-scale naloxone program, though only significant among men [18]. This highlights the need for further research into how structural vulnerability and gendered dispositions shape and support needs across the life course among PWUD.

Even before naloxone became available to lay persons, individuals who themselves use drugs have been the first line of responders regardless of who, friends, family members and strangers have suffered from overdose [85, 86]. Getting involved in overdose situations which resembled previous traumatic incidents reportedly fueled anxiety and made participants re-live trauma, which quite a few struggled with emotionally. Even though this was expressed by several participants, all denied any hesitation in engaging in future overdose situations. Previous findings describe a high degree of burnout and stress among EMS personnel [87–89]. Most Supersavers face the same stress, anxiety and trauma, with the additional emotional layer of the victim often belonging to their own social network of friends or family members. This matches previous findings on peer overdose intervention internationally [33, 72]. Although often being the first responder to overdose, either while waiting for EMS to arrive, or instead of [19, 69], research has shown extensive barriers in accessing both mental health and other support services for individuals suffering from co-occurring substance use disorder and mental health disorders [90].

The collective sense of responsibility and compassion demonstrated by Supersavers has been well-documented among peers engaging in OEND internationally [28–31, 33, 58, 91–93]. In this study, most participants framed their involvement as a way of affirming self-worth and demonstrating that they were trustworthy, competent, and genuinely caring for others. In doing so, they actively challenged stigmatizing and reductive stereotypes frequently ascribed to PWID [28, 94].

The ability to respond effectively to overdoses and care for others may constitute a form of symbolic or “street” capital [49], conferring recognition and value among peers. Acts of helping are thus not only altruistic, but also meaningful practices through with individuals establish competence, moral worth and belonging. Naloxone, in this context, was often described as a resource that enhanced participants’ capacity to act, increasing both perceived safety and their ability to fulfil these roles.

At the same time, this position may come at a cost. Previous research has shown that repeated exposure to overdose situations can lead individuals to withdraw from social ties due to stress and the burden of responsibility becoming too great [28, 58, 95]. While this suggests the possibility of disengagement, of having a “way out”, several participants in this study, particularly older individuals, described the opposite, expressing a sense of having no viable alternative but to continue. This reflects a form of entrapment within the field, where accumulated experiential capital and moral expectations reinforce continued involvement.

Close parallels can be drawn to what has been described as “trap life” adaptation, where repetitive trauma, incarceration, and premature death become embedded features of everyday life, with limited opportunities of getting out [96]. Within such contexts, the normalization of overdose and repeated exposure to crisis may contribute to desensitization, potentially diminishing recognition of the need for support. From a risk environment perspective, these patterns also reflect broader structural vulnerabilities, where limited resources, stigma and marginalization constrain both opportunities for disengagement and access to care. Together, these findings highlight how responsibility and compassion are embedded within social relations that both enable and constrain action, producing forms of value, but also reinforcing cycles of vulnerability.

While most participants expressed how it was “too late” for them to resolve their traumas, they emphasized the importance of targeting particularly vulnerable groups. In correlation to previous findings by Song et al., several participants described heightened trauma during their first experience reversing an overdose [32]. Supported by previous research [97, 98], Supersavers worried that overdose awareness among young opioid users was low and that they were very naive, despite their elevated risk of both non-fatal and fatal opioid overdose. Young opioid users were commonly described by participants as reckless and uninformed, casually using prescription opioids like “oxy” (oxycodone) and “tram” (tramadol), believing these to be “clean” or safer than other opioids, often underestimating the addictive potential and risks associated with these drugs. Study participants therefore suggested prioritizing outreach services targeting novice users “early in the process”, as this could help prevent accumulation of trauma. Beyond novice users, undocumented young migrants were also highlighted as an especially vulnerable population. Participants noted that fear of deportation often prevented this group from accessing social and healthcare services. Despite legal barriers limiting peer-based education and naloxone distribution, many Supersavers expressed a strong sense of responsibility to reach individuals beyond the scope of formal services through peer networks. While this highlights unmet needs, it also reinforces structural vulnerabilities as it relies on individuals with lived experience of stigma and systemic exclusion operating as informal healthcare providers, acting as intermediaries between a distrusted system and those who, for different reasons, remain disengaged from it. Thus, it highlights the potentiality of community-based distribution, provided that their involvement is formalized, and that peer engagement and hard work is met by recognition, support, equitable pay and benefits equivalent to that of other healthcare professionals.

Simply implementing naloxone programs will not change the structuring structures and power-relationships practices. Viewed through the ‘risk environment’-lens, the everyday practices of PWUD are heavily constrained, forcing them to conceal their use and to consume drugs in unsafe or isolated settings, facing limited or no options. Though behavioral interventions promoting safer drug practices remain imperative, they should be accompanied by efforts to address the broader structural and systemic barriers that perpetuate drug-related harm [59]. For Supersavers continued engagement, sustainable conditions and sufficient and appropriate support need to be ensured. Their vital contributions should not come at the cost of their own wellbeing; efforts must be met with tailored support that aligns with their needs and realities. Structural problems demand structural solutions. These results imply a need for further research not only to acknowledge and understand the complex structures which impede and enable practices of overdose management but also point to the importance of studying the different gendered conditions. It also highlights the importance of providing highly available and accessible support, and from a structural perspective it inevitably forces us to ask the question whether we are dealing with hard-to-reach healthcare and social welfare instead of hard-to-reach individuals.

## Strengths and limitations

Several limitations should be considered. First, overdose reversals were based on self-report and may be subject to misclassification. Within the program, participants can receive new naloxone kits regardless of how prior use is reported (e.g., lost, given away, or used), which may reduce the incentive for precise reporting and introduce uncertainty in reversal counts. Underreporting is therefore possible, particularly given the sensitive nature of overdose events and potential concerns about stigma or unwanted attention.

Second, the “three or more” reversal threshold used to define “supersavers” was based on prior Scandinavian literature and the distribution of reported reversals at the time of recruitment. However, our findings indicate that many participants reported substantially higher numbers of reversals, suggesting that this cutoff may be conservative. As naloxone programs expand and mature, individuals with repeated overdose response experience may become more common, and fixed thresholds may not fully capture the upper range of community response activity.

Additionally, data was based on self-report which may be affected by recall bias, social desirability bias and reporting errors. The relatively small sample were recruited from the largest NSP program in South Sweden, which may imply that Supersavers experiences may differ from that of the broader group of individuals with less overdose experience, or those with other patterns in opioid use. Supersavers experiences do not represent all Malmö NSP visitors, nor those engaged in reversals not visiting the facility which may inherently limit the wider transferability of the observations. Observations in differences between males and females within the group may also differ to that of the larger population. Nevertheless, these observations are important as the rather small group of Supersavers does reverse a large proportion of overdoses within their communities. It is vital to understand their situation and their experiences if we want to be able to offer sufficient support for them to continue in their engagement.

A further limitation of this study is that participants’ accounts largely emphasized successful overdose reversals and positive aspects of their involvement. This may reflect the purposive sampling of individuals with repeated overdose response experience, but it may also be shaped by the interview context. As highlighted in narrative research [52], interview narratives may involve forms of self-presentation that emphasize competence and responsibility. Consequently, more challenging or unsuccessful experiences may be underrepresented and should be considered when interpreting the findings.

## Conclusions

This study shows how Supersavers’ practices emerge through the interplay of Bourdieus’ habitus, capital and field within structurally constrained contexts of Rhodes’ risk environment framework. Repeated overdose reversals have produced an embodied habitus characterized by rapid recognition, intervention and a strong sense of responsibility for others. This is underpinned by “street capital” in the form of experiential knowledge, technical skills and peer trust.

Naloxone expanded this capital, reducing the physical and emotional burden of reversals and enabling more effective risk mitigation, including for their own overdose vulnerability. At the same time, frequent exposure to overdose contributed to normalization and potential desensitization, while sustaining fears of losing others. Gendered differences in risk perception and access to support further shaped engagement.

Despite pride and empowerment in their role, Supersavers operate within a risk environment marked by limited trust in formal services, leading peers to rely on them over emergency care. Their continued engagement is sustained through responsibility and experience yet strained by cumulative trauma and unmet support needs. Strengthening harm reduction requires not only resources, but accessible, context-sensitive healthcare support to sustain this critical workforce.

## Supplementary Information

Below is the link to the electronic supplementary material.

Supplementary Material 1

## Data Availability

The data used to support the findings of this study are restricted by the Regional Ethics Board, Lund, Sweden, to protect patient privacy. Data are available from Katja Troberg, katja.troberg@med.lu.se for researchers who meet the criteria for access to confidential data.

## Abbreviations

EMS: Emergency Medical Services
NSP: Needle and Syringe Program
OAT: Opioid Agonist Therapy
OEND: Opioid Education and Naloxone Distribution
PWID: People Who Inject Drugs
PWUD: People Who Use Drugs
THN: Take-Home Naloxone
