# Supplementary material for: “You’ve involved us knowing that we don’t think about the law like you”: a qualitative study on Supersavers’ perspective of overdose engagement in Skåne county, South Sweden
Source: Harm Reduct J. 2026 Jun 23;23:112. doi: 10.1186/s12954-026-01487-x (PMC13307409; doi:10.1186/s12954-026-01487-x)
Supplement: Supplementary file 1 — Supplementary Material 1 [file 12954_2026_1487_MOESM1_ESM.docx]

Additional file 1.

| Table 1. Participant characteristics (n=13) | | | | |
| --- | --- | --- | --- | --- |
| Participant | Gender | Age group | Number of overdose reversals with naloxone, reported during NSP refill (n=71) | Number of over­dose reversals with naloxone, reported during interview (n=131) |
| 1 | Female | 45–49 | 5 | 10 |
| 2 | Male | 40–44 | 3 | 10 |
| 3 | Male | 45–49 | 3 | 4 |
| 4 | Female | 30–34 | 4 | 4 |
| 5 | Female | 55–59 | 14 | 22 |
| 6 | Male | 30–34 | 4 | 12 |
| 7 | Male | 45–49 | 6 | 7 |
| 8 | Female | 40–44 | 7 | 10 |
| 9 | Male | 50–54 | 5 | 30 |
| 10 | Male | 40–44 | 4 | 6 |
| 11 | Female | 30–34 | 3 | 3 |
| 12 | Male | 60–64 | 7 | 7 |
| 13 | Male | 30–34 | 6 | 6 |
| Mean | 38% female | 43.5 | 5.5 | 10.1 |
